# Evaluating the Efficacy of Brensocatib in Non-cystic Fibrosis Bronchiectasis

**DOI:** 10.7759/cureus.117538

**Published:** 2026-10-06

**Authors:** Alan Mathew, Hannah Anwar

**Affiliations:** 1 Internal Medicine, Basildon and Thurrock University Hospitals NHS Foundation Trust, Basildon, GBR; 2 Medical Education, Basildon and Thurrock University Hospitals NHS Foundation Trust, Basildon, GBR

**Keywords:** brensocatib, dpp-inhibitors, non-cf bronchiectasis, polypharmacy, review of the literature

## Abstract

Bronchiectasis is a widely recognised chronic respiratory illness classified in severity by frequency of exacerbations within the past year, percent predicted forced expiratory volume in 1 second (%FEV1), previous hospital admissions and radiological severity, among other factors. The physiological process is understood to be driven by neutrophilic inflammation and subsequent bronchial wall destruction and dilatation. Brensocatib has emerged as a novel adjunct in the management of chronic bronchiectasis as a dipeptidyl peptidase-1 inhibitor, directly targeting the inflammatory process. The editorial aims to review the mechanism of action of brensocatib, current literature surrounding its efficacy and side effect profile, and potential implications of a wider rollout of the medication to the adult UK population. Whilst the current literature does support a modest reduction in the frequency of exacerbations for patients who trialled bresnocatib, there is a large amount of uncertainty regarding long-term safety, implementation into the current regime, and cost-effectiveness.

## Editorial

Bronchiectasis is a chronic respiratory disorder characterised by the abnormal dilation of bronchi due to the destruction of both elastic and muscular components of the bronchial wall [1]. It is caused by airway inflammatory changes due to recurrent lower respiratory tract infections [2] and has a higher prevalence in women and older individuals [3]. Between 2004 and 2013, the prevalence of bronchiectasis in the UK rose from 350.5 per 100,000 to 566.1 per 100,000 in women and 301.2 per 100,000 to 485.5 per 100,000 in men [4]. Current non-pharmacological treatments for bronchiectasis emphasise exercise, improved nutrition, airway clearance therapies and consideration of mucoactive agents [1] to reduce exacerbations and focus on symptom management, with the addition of macrolide antibiotics to mitigate the inflammatory process. A recent meta-analysis conducted in 2026 addressing 24 studies involving 7,051 patients found that mucoactive therapies reduced exacerbations by 0.40 per year; however, the confidence interval means that more data are required to further assess reduction in exacerbation rates [5]. In February 2026, the Medicines and Healthcare Products Regulatory Agency (MHRA) authorised brensocatib (Brinsupri) as the first medical treatment designed to treat non-cystic fibrosis bronchiectasis in patients 12 years and older who have experienced worsening symptoms or two or more flare-ups in the past 12 months [6]; this follows the approval from the European Commission and the U.S. Food and Drug Administration the year prior. Brensocatib is an oral inhibitor of the enzyme dipeptidyl peptidase-1, which activates neutrophil serine proteases [7]. Targeting neutrophil-led inflammation offers a potential approach to improve outcomes in patients with non-cystic fibrosis bronchiectasis by directly targeting the inflammatory pathway. However, the significance of the clinical benefits needs to be weighed against the long-term implications and potential adverse effects. 

Neutrophilic inflammation is characteristic of bronchiectasis, with chronic infection, airway inflammation, impaired mucociliary clearance and structural lung damage making up a ‘vicious vortex’ [8]. Bacterial infection stimulates the recruitment of inflammatory cells, specifically neutrophils, to fight the infection, perform phagocytosis of bacteria, degranulation, reactive oxygen species production and formation of neutrophil extracellular traps (NETs), which are associated with severe bronchiectasis [8]. This process leads to tissue damage in the lungs, and impaired mucus clearance encourages further infection, perpetuating the cycle. Chalmers et al.’s study found that patients with a neutrophil elastase >20 µg/mL had a 3.18-fold higher rate of exacerbations and were also associated with a higher severity of bronchiectasis and lung function decline [9]. The dipeptidyl peptidase-1 enzyme is responsible for activating the main neutrophil serine proteases, including neutrophil elastase (NE), proteinase 3 (PR3) and cathepsin G (CatG), and brensocatib is an oral drug that directly inhibits dipeptidyl peptidase-1. This results in a reduction in the amount of active protease carried by neutrophils, which are recruited. A 24-week phase 2 trial in 256 patients (WILLOW) of brensocatib in different strengths against a placebo shows that the 25^th^ percentile for time to first exacerbation was 67 days in patients on the placebo, 134 days in patients on 10 mg, and 96 days in patients on 25 mg of brensocatib [10], demonstrating a clinical improvement in patients. However, the adjusted hazard ratios for exacerbation in comparison of bresnocatib with the placebo were 0.58 and 0.62, respectively, in the 10 mg and 25 mg groups, highlighting the increased incidence of dental and skin adverse effects compared to the placebo [10]. A Phase 3 trial (ASPEN) also concluded that in a total of 1,721 patients, the annual rate of exacerbations was 1.29 in the placebo group, 1.02 in the group on 10 mg and 1.04 in the group on 25 mg [11]. With regard to long-term damage, the 25 mg dose showed a slower decline in forced expiratory volume in 1 second (FEV1) compared to placebo (38 ml over 52 weeks), whilst the 10 mg dose did not. The above data suggest that severe exacerbations of bronchiectasis, thus requiring hospital admission, were not significantly reduced, a significant factor that should be considered by the National Institute for Health and Care Excellence (NICE) for a potential wide rollout in the UK. 

Despite the promising findings found in the clinical trials, more data are required to thoroughly evaluate long-term outcomes in all patient groups. For example, the ASPEN trial did not evaluate the effectiveness of bresnocatib in patients with milder disease, as the enrolment criteria were patients with ≥ 2 exacerbations per year [12]. Further research is also required to assess the potential long-term implications of side effects on patients’ quality of life, as the respective scores for the 10mg and 25mg doses were increased by 2 and 4 points in the Quality-of-Life Bronchiectasis (QOL-B) questionnaire (compared to placebo), although this score was only used for baseline assessment [13]. Common adverse effects include gastroenteritis, upper respiratory tract infections, headache, hyperkeratosis and gingival disorders as outlined in the European Medicines Agency brencosatib summary [14]. It is worth noting that there has been an established link with the absence mutations resulting in the loss of dipeptidyl peptidase-1, culminating in Papillon-Lefèvre syndrome, which is characterised by hyperkeratosis and gingival changes [10]. Consideration of efficacy in all risk groups and evaluation of long-term adverse effect prevalence are pertinent when reviewing the established foundations of bronchiectasis management. For example, only 12 adolescents were enrolled in the ASPEN trial; however, the MHRA authorised bresnocatib for patients 12 years and older. As such, further clinical trials should be held to assess this patient demographic. With regard to cost-effectiveness, the US estimates the value of bresnocatib at ~$88,000 per quality-adjusted life year gained for the 25 mg dose, far higher than the projected $3100-$3700 per year threshold to meet cost-effectiveness [12]. NICE would need to decide whether bresnocatib’s modest benefit would justify this significant cost at launch. If NHS funding were approved for commercial supply, patients should be reminded that brencosatib should not function as a substitute for known effective strategies such as airway physiotherapy, management of underlying asthma/chronic obstructive pulmonary disease (COPD) and long-term antibiotic therapy (if indicated) [13]. It is important to note that bresnocatib’s effect on reduction in frequency of exacerbations was similar regardless of whether patients had been established on macrolide therapy [15]. Moreover, the impact of polypharmacy must not be understated when reviewing long-term patient outcomes. Research has concluded that bronchiectasis medication regimens consisting of 10 or more medications have been associated with poorer outcomes for patients, resulting in more frequent hospital admissions for IV antibiotic therapy [16]. Therefore, particular emphasis must be placed on the evaluation of current medication regimens and frequency of admissions for patients before brensocatib can be considered as adjunct therapy. Currently, widespread NHS availability is subject to the approval of NICE [17]. Once commercial supply is approved, the NHS must have bronchiectasis clinics and the Yellow Card scheme to conduct post-marketing surveillance and maintain pharmacovigilance with regard to potential long-term complications of adverse effects. 

Overall, the results of clinical trials such as ASPEN and WILLOW demonstrated a reduction in the frequency of bronchiectasis exacerbations and an increase in the amount of time until the first exacerbation, respectively, in patients with non-cystic fibrosis bronchiectasis with more frequent admissions. However, further research is required with at least another Phase 2 or 3 clinical trial with a large sample size targeting patients with moderate non-cystic fibrosis bronchiectasis. Emphasis also needs to be placed on perceived improvements to patients’ quality of life, as the data collated in this parameter were solely for baseline assessment and not for significance testing. Given the current pharmaceutical burden associated with established bronchiectasis treatment regimens, further research needs to be conducted on whether brensocatib can reduce the need for prophylactic antimicrobials and thus mitigate polypharmacy. Despite the MHRA licensing brensocatib under specific treatment parameters, it has not been formally approved by NICE for a wide rollout in primary and secondary care settings. In conclusion, further extensive research must be conducted on bresnocatib with regard to its cost-effectiveness, impact on more patient risk groups and whether it can be used as an adjunct/replacement to existing macrolide therapies. 
